# Change of perspective: impact of COVID-19 pandemic on axial spondyloarthritis-related web searches in Germany

**DOI:** 10.1038/s41598-024-54047-3

**Published:** 2024-02-19

**Authors:** Hannah Wecker, Stefanie Ziehfreund, Michael Hindelang, Martin Welcker, Alexander Zink

**Affiliations:** 1 Department of Dermatology and Allergy, TUM School of Medicine and Health, Technical University of Munich, Munich, Germany; 2 Pettenkofer School of Public Health, Munich, Germany; 3 Institute for Medical Information Processing, Biometry, and Epidemiology-IBE, LMU Munich, Munich, Germany; 4 Medizinisches Versorgungszentrum für Rheumatologie Dr. M. Welcker GmbH, Planegg, Germany; 5 RheumaDatenRhePort GbR, Planegg, Germany; 6 Division of Dermatology and Venereology, Department of Medicine Solna, Karolinska Institutet, Stockholm, Sweden

**Keywords:** Spondyloarthritis, Epidemiology, Skin diseases

## Abstract

Several conventional cross-sectional studies have investigated the impact of the coronavirus disease (COVID-19) pandemic on patients with axial spondyloarthritis (axSpA) and reached contrary results regarding health and well-being. As analysis of web search data already provided insights into public interest and unmet needs, this study aimed to examine axSpA-related web searches before and during COVID-19 pandemic to gain a different perspective on the impact of COVID-19 on this disease. The Google Ads Keyword Planner was used to generate axSpA-related keywords and their monthly number of searches between June 2018 and November 2021 in Germany. These keywords were qualitatively classified into seven categories. A total of 538 axSpA-related keywords were used for the analysis. The number of axSpA-related searches increased during COVID-19 pandemic (before: n = 1,525,010 vs. during: n = 1,848,300), particularly searches for symptoms, disease outcomes, and causes, while interest in disease management and diagnosis decreased. This study demonstrated a shift in public interest in axSpA during COVID-19 in Germany and highlights an urgent expansion of telemedicine to be prepared for exceptional situations such as a pandemic.

## Introduction

Axial spondyloarthritis (axSpA) is a chronic inflammatory disease that primarily affects the sacroiliac joints and the spine with inflammatory back pain, but can also include extra-articular manifestations like arthritis, enthesitis, psoriasis, iritis, and inflammatory bowel disease^1^. In March 2020, the World Health Organization declared a global pandemic caused by coronavirus disease (COVID-19)^2^, initiating a period of restrictions and multiple lockdowns, which could lead to new diseases or exacerbation of pre-existing diseases by reducing healthcare provider consultations and/or affecting mental health^3,4^. Ciuera et al. found no evidence of worsening disease activity in axSpA during the lockdown^5^, while others demonstrated that approximately 50% of axSpA patients reported worse health status and well-being than before the pandemic^6,7^. As analysing web search data on health topics can contribute to a better understanding of the interest and needs on a population-level^8,9^, this study aimed to examine the impact of the COVID-19 pandemic on axSpA in Germany by comparing axSpA-related web searches before and during the COVID-19 pandemic (cut-off: March 2020) in terms of search content.

## Methods

**Figure 1:**
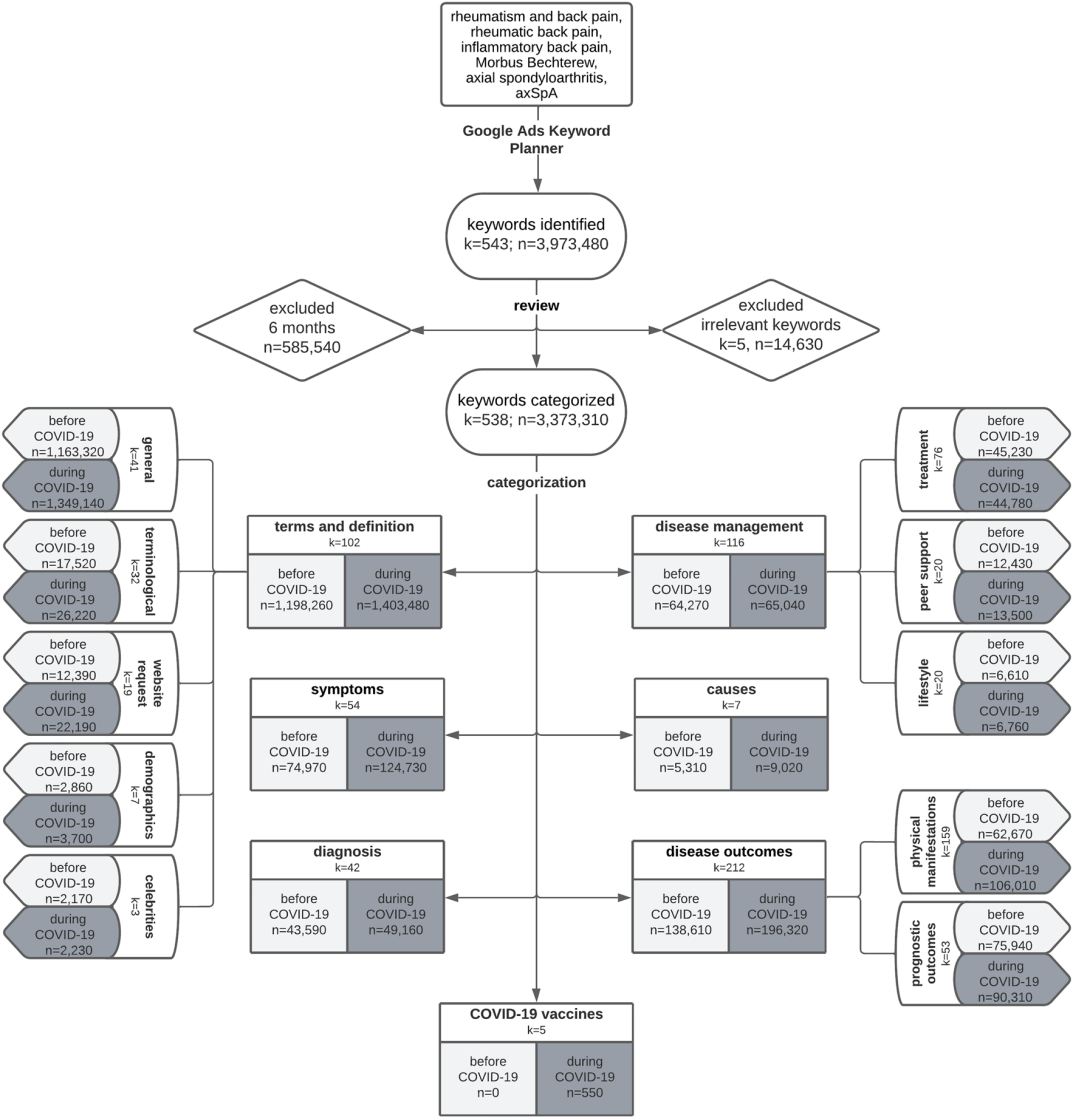
Flowchart of data generation and content categorisation. The axSpA-related web searches in Germany before (06/2018–02/2020) and during COVID-19 pandemic (03/2020–11/2021) were analysed. *axSpA* axial spondyloarthritis, *COVID-19* coronavirus disease, *k* number of keywords, *n* number of searches.

In this retrospective analysis, the Google Ads Keyword Planner was used. Originally designed for marketing campaigns, its capability to provide monthly search volume data (i.e., monthly number of web searches)^8,10^. To ascertain the search volume in a specific area, search terms are input into the planner, language and geographical settings are set, and the program subsequently generates the most relevant keywords and phrases for the topic. For this study, six German search terms were entered to obtain related keywords and phrases with their monthly search volume between June 2018 and May 2022 in Germany (Fig. 1). The n = 543 identified keywords were individually checked for their relevance to axSpA, leading to the exclusion of five keywords which were unspecific and not clearly axSpA-associated (e.g., “back pain due to bacteria”, “inflamed back muscle“). The time period was shortened by 6 months to ensure an equal number of months before and during COVID-19. The remaining keywords were inductively categorised into seven categories based on the study by Berr et al.^8^: (1) *terms and definition* (e.g., “spondyloarthritis”, “rheumatism Morbus Bechterew”), (2) *disease outcomes* (e.g., “life expectancy”, “rheumatism back pain”), (3) *disease management* (e.g., “treatment”, “experience reports”), (4) *diagnosis* (e.g., “Bechterew diagnosis”, “HLA B27”), (5) *symptoms* (e.g., “rheumatism back symptoms”), (6) *causes* (e.g., “Morbus Bechterew causes”), and (7) *COVID-19 vaccines*. For recurring topics, categories were further classified into subcategories (e.g., *physical manifestations*). Each keyword was assigned to one (sub-)category only. Data was analysed descriptively.

### Ethical approval

Institutional review board approval and informed consent were not necessary, because the study used publicly available data.

## Results

**Figure 2:**
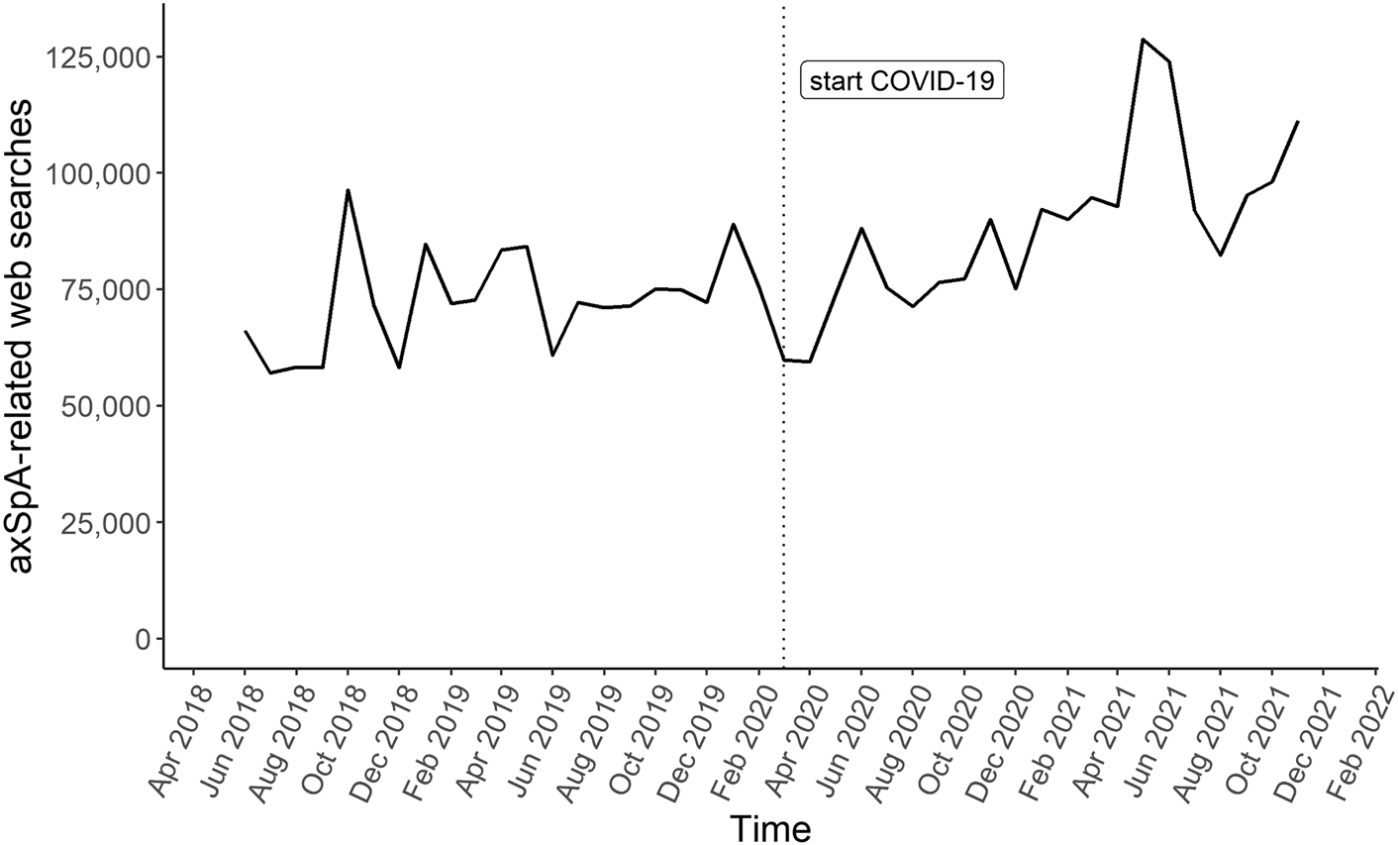
Time course of axSpA-related web searches from June 2018 to November 2021 in Germany. axSpA, axial spondyloarthritis.

**Figure 3:**
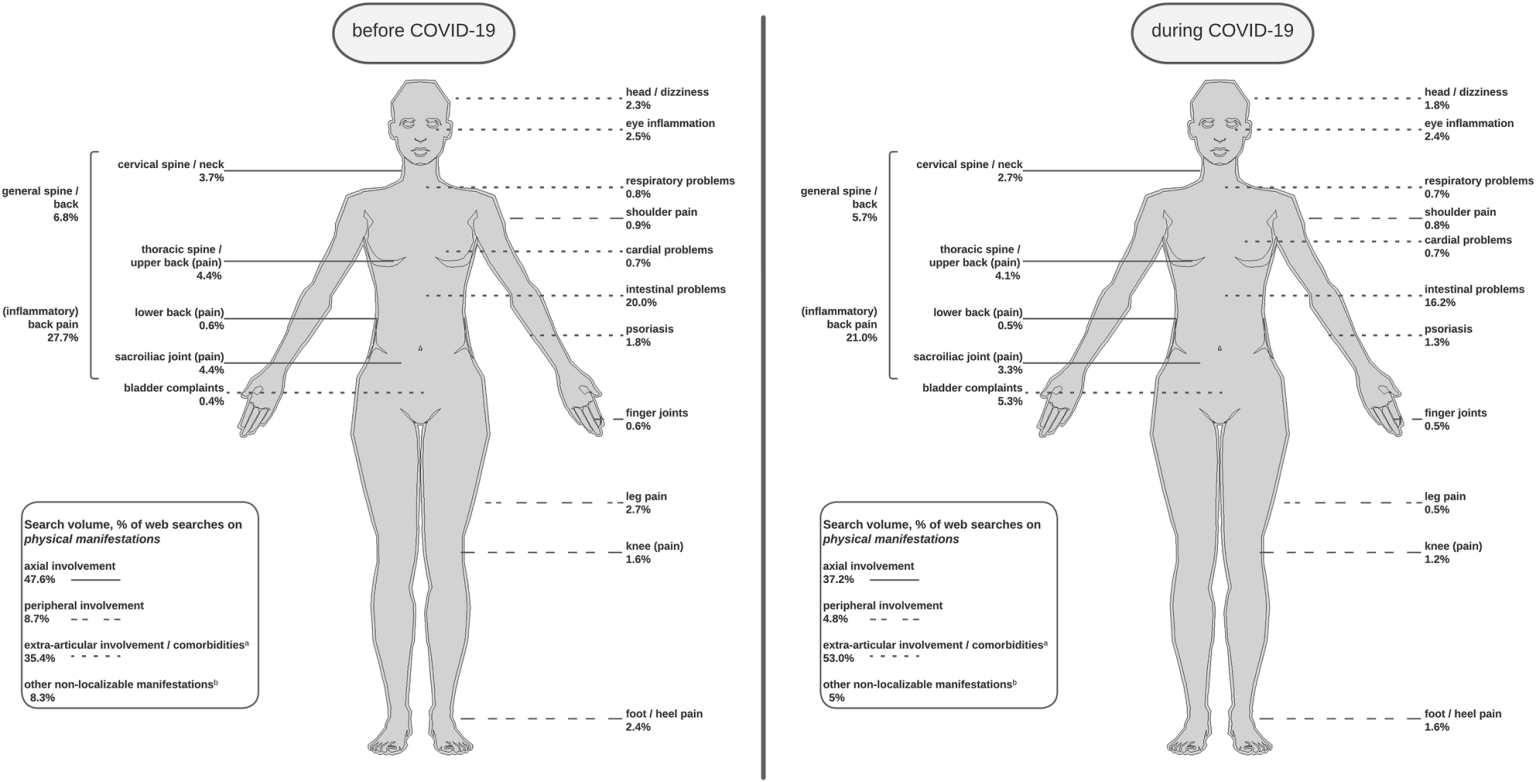
Physical manifestations in axSpA-related web searches. Percentage of axSpA-related web searches on subcategory *physical manifestations* of axSpA in Germany before (06/2018–02/2020) and during COVID-19 pandemic (03/2020–11/2021). (**a**) Including non-localisable and general searches for comorbidities (before COVID-19: 15.8%, during COVID-19: 29.8%; e.g., “comorbidities morbus bechterew” or “weight loss morbus bechterew”). (**b**) E.g., “morbus bechterew pain”. The contour of the female body was obtained from the R package “gganatogram” developed by Maag JLV (2018): doi: https://doi.org/10.12688/f1000research.16409.2. axSpA, axial spondyloarthritis; COVID-19, coronavirus disease.

Overall, 538 keywords with n = 3,373,310 axSpA-related searches were identified for Germany between June 2018 and November 2021 using Google Ads Keyword Planner (Fig. 1). Although axSpA-related searches were at a low level at the beginning of the COVID-19 pandemic (Fig. 2), searches increased during the COVID-19 pandemic (before: n = 1,525,010 vs. during: n = 1,848,300). For both periods, “Morbus Bechterew” (German synonym for axSpA) was most frequently searched and about three-quarters of all searches were related to *terms and definitions*, which was in line with previous findings^8^. During COVID-19, axSpA searches related to *symptoms* (4.9% vs. 6.7%), *disease outcomes* (9.1% vs. 10.6%), and *causes* (0.3% vs. 0.5%) slightly increased, whereas searches decreased for *disease management* (4.2% vs. 3.5%) and *diagnosis* (2.9% vs. 2.7%, Fig. 1). Searches on *disease outcomes* shifted from *prognostic outcomes* (54.8% vs. 46.0%) to *physical manifestations* (45.2% vs. 54.0%), with higher interest in *comorbidities and extra-articular involvement* (35.4% vs. 53.0%) than in *axial involvement* (47.6% vs. 37.2%, Figs. 1, 3). Searches on axSpA *prognostic outcomes*, particularly *life expectancy*, decreased during COVID-19 (50.2% vs. 35.7%), while searches for *end stages* of axSpA (18.3% vs. 22.8%), *course of disease* (13.1% vs. 19.9%), and *work disability* (4.8% vs. 9.1%) increased. Among *disease management* of axSpA, searches slightly differed between before and during COVID-19: searches related to *treatment* (70.4% vs. 68.8%) decreased and searches related to *lifestyle* (10.3% vs. 10.4%) and *peer support* slightly increased during COVID-19 (19.3% vs. 20.8%, Fig. 1).

## Discussion

In summary, the initial decrease in axSpA-related searches during the pandemic could be explained by the widespread attention regarding COVID-19^10^. However, overall axSpA-related searches increased during COVID-19, particularly for *symptoms* and *physical manifestations*, in addition to unknown factors not attributable to the pandemic, possibly due to the lack of medical appointments to clarify symptoms, provide medical information, and support for those affected. A reduction of consultations in German primary care during COVID-19 was previously found, mainly concerning manifestations of axSpA like back pain, gastrointestinal symptoms, fatigue, and pain therapy^1,3^, which may have led to the exacerbation of axSpA^7^. The higher interest in poor disease outcomes, like *end stage* and *work disability*, and in *peer support* may reflect general mental health impairments during lockdowns in the German population^4^, warranting further exploration. Additionally, the observed emphasis on these aspects may be attributed to the lack of information and comfort during restrictions resulting from the absence of in-person rheumatology appointments, which were rated as very important by the majority of axSpA patients surveyed in the United Kingdom^7^. Despite limitations, including the focus on Google users, exclusion of non-German speakers and potentially elderly, the impact of autocomplete, and reliance on monthly estimations, the study highlights the change in unmet needs. This emphasizes the urgency need to expand telemedicine concepts and increase patient confidence in them, to provide healthcare in exceptional situations, like a global pandemic.

## Data Availability

The datasets generated during and/or analysed during the current study are available from the corresponding author on reasonable request.
